# Profiling inflammatory outcomes of *Candida albicans* colonization and food allergy induction in the murine glandular stomach

**DOI:** 10.1128/mbio.02113-24

**Published:** 2024-09-30

**Authors:** Karen D. Zeise, Nicole R. Falkowski, Kelsey G. Stark, Christopher A. Brown, Gary B. Huffnagle

**Affiliations:** 1 Department of Microbiology & Immunology, University of Michigan, Ann Arbor, Michigan, USA; 2 Mary H. Weiser Food Allergy Center, University of Michigan, Ann Arbor, Michigan, USA; 3 Department of Molecular, Cellular, and Developmental Biology, University of Michigan, Ann Arbor, Michigan, USA; 4 Division of Pulmonary & Critical Care Medicine, University of Michigan, Ann Arbor, Michigan, USA; 5 Advanced Research Computing, Information and Technology Services, University of Michigan, Ann Arbor, Michigan, USA; Geisel School of Medicine at Dartmouth, Hanover, New Hampshire, USA

**Keywords:** *Candida albicans*, food allergy, stomach, immunomodulation, ovalbumin

## Abstract

We investigated the effects of *Candida albicans* colonization on inflammatory responses in the murine glandular stomach, which is similar to the glandular mucosa of the human stomach. We also explored whether the presence of a food allergy could exacerbate *C. albicans*-induced inflammation or if *C. albicans* would amplify allergic inflammation in the glandular stomach. *C. albicans* successfully colonized the stomach of amoxicillin-pre-treated BALB/c mice and induced gastritis in the limiting ridge with minimal inflammation in the glandular stomach. There was significant upregulation of *Il18*, calprotectin (*S100a8* and *S100a9*), and several antimicrobial peptides, but minimal induction of type 1, 2, or 3 responses in the glandular stomach. A robust type 2 response, inflammatory cell recruitment, and tissue remodeling occurred in the glandular stomach following oral ovalbumin challenges in sensitized mice. The type 2 response was not augmented by *C. albicans* colonization, but there was significant upregulation of *Il1b*, *Il12a*, *Tnf*, and *Il17a* in *C. albicans*-colonized food allergic mice. The presence of *C. albicans* did not affect the expression of genes involved in barrier integrity and signaling, many of which were upregulated during food allergy. Overall, our data indicate that *C. albicans* colonization induces minimal inflammation in the glandular stomach but augments antimicrobial peptide expression. Induction of a food allergy results in robust type 2 inflammation in the glandular stomach, and while *C. albicans* colonization does not exacerbate type 2 inflammation, it does activate a number of innate and type 3 immune responses amid the backdrop of allergic inflammation.

**IMPORTANCE:**

Food allergy continues to be a growing public health concern, affecting at least 1 in 10 individuals in the United States alone. However, little is known about the involvement of the gastric mucosa in food allergy. Gastrointestinal *Candida albicans* colonization has been reported to promote gastrointestinal inflammation in a number of chronic diseases. Using a mouse model of food allergy to egg white protein, we demonstrate regionalization of the inflammatory response to *C. albicans* colonization, induction of robust type 2 (allergic) inflammation in the stomach, and augmentation of innate and type 3 responses by *C. albicans* colonization during food allergy.

## INTRODUCTION

*Candida albicans* is a ubiquitous fungal colonizer of humans, residing predominantly in the oral cavity, gastrointestinal tract, and vaginal mucosa, and can readily colonize the gastrointestinal tract of inbred mice after experimental introduction (1, 2). Within the stomach, *C. albicans* can exist as a non-inflammatory commensal or induce considerable inflammation. *C. albicans* colonization is well documented in the stomach of both humans and rodents, where an extremely low pH and high concentration of proteolytic enzymes restrict most microbial growth (3–7). However, disruption of the bacterial microbiota and/or host immunity can trigger a pathogenic transformation, leading to significant local infection as well as systemic dissemination. A dynamic cell wall—including the ability to switch between yeast and hyphal morphology—enables *C. albicans* to readily adapt to diverse host microenvironments and exploit immune defenses to favor its survival (8–10). In addition, *C. albicans* possesses a plasma membrane H^+^-ATPase, which facilitates its growth in niches ranging from highly acidic to alkaline (7). In humans, *C. albicans* can incite or exacerbate chronic gastritis and peptic ulcerous disease (6, 11, 12), and the use of acid-suppressing therapy (including proton pump inhibitors and histamine-2 receptor antagonists) is a known risk factor for the development of gastroesophageal candidiasis (11).

The murine stomach has three anatomically distinct regions: forestomach, limiting ridge, and glandular stomach (“hindstomach”) (13). The epithelium of the forestomach is comprised of a keratinized stratified squamous epithelium, similar to that in the human esophagus. In contrast, the epithelium of the glandular stomach is comprised of glandular columnar epithelium, which more closely resembles that of the human stomach. At the junction between these regions is the limiting ridge, which is an outcropping of the epithelium (covered by stratified squamous epithelium) that encircles the internal lining of the stomach. While the function of the limiting ridge is unknown, it represents the physical junction between the forestomach and glandular stomach and likely plays a role in regulating the passage of food for digestion. One notable feature of *C. albicans*-induced gastritis in the murine stomach is that it is almost exclusively localized to the limiting ridge (3, 14–19). In contrast to the limiting ridge, the glandular stomach epithelium during *C. albicans* infection appears to be histologically devoid of inflammatory infiltrates, raising the possibility that the model described earlier of the gastric mucosal response to *C. albicans* colonization may largely reflect the host response in the limiting ridge but not in the glandular stomach. In other words, the immune response in the murine stomach may be regionalized, and the response in the glandular stomach may be distinct from that in the limiting ridge. Thus, our objective was to analyze the host response in the glandular stomach during *C. albicans* colonization of the stomach.

In addition, we investigated whether the induction of a food allergy will augment *C. albicans* pathogenesis and inflammation in the glandular stomach and whether *C. albicans* colonization augments the food allergic response. Our laboratory previously demonstrated that gastrointestinal colonization of either BALB/c or C57BL/6 mice with *C. albicans* can promote type 2-polarized allergic disease in distal tissues, such as the airways (20, 21). In mouse models of dextran sodium sulfate-induced colitis, *C. albicans* augments inflammation and exhibits increased growth (22, 23). These findings suggest a “feed-forward” mechanism in which an inflammatory environment enables *C. albicans* to become pathogenic, which further enhances mucosal inflammation and may involve the direct effects of IL-17A and aryl hydrocarbon receptor metabolites on the fungus (24, 25). *C. albicans* colonization of mice has also been shown to inhibit the induction of oral tolerance for humoral immune responses (26). Studies in BALB/c mice have shown that gastrointestinal permeation of the food antigen ovalbumin (OVA) is enhanced by *C. albicans* colonization and can promote sensitization to food antigens through a mast cell-mediated mechanism (27). Subsequent studies in C57BL/6 mice have demonstrated a similar effect of *C. albicans* colonization on gastrointestinal barrier function, with IL-9 induction of TGF-β in stromal mast cells (MCs) of the stomach and small intestine, and modulation of indoleamine 2,3-dioxygenase activity (28). In light of the recent report showing gastric mucosal inflammation in individuals with eosinophilic gastritis/gastroenteritis (29), there is a need for a more comprehensive analysis of the gastric mucosal immune response in food allergy.

Thus, the objectives of our present study were to investigate (i) the mucosal response to *C. albicans* within the glandular stomach, (ii) the mucosal response to food allergy induction in the glandular stomach, and (iii) whether the gastrointestinal colonization with *C. albicans* affects the mucosal response in the glandular stomach during food allergy. We evaluated the expression of more than 80 genes associated with a variety of mucosal immune and epithelium responses, focusing our analysis on the glandular region of the murine stomach because it most closely resembles the glandular mucosa of the human stomach (13).

## RESULTS

### *Candida albicans* colonization and host response in the murine glandular stomach

**Fig 1:**
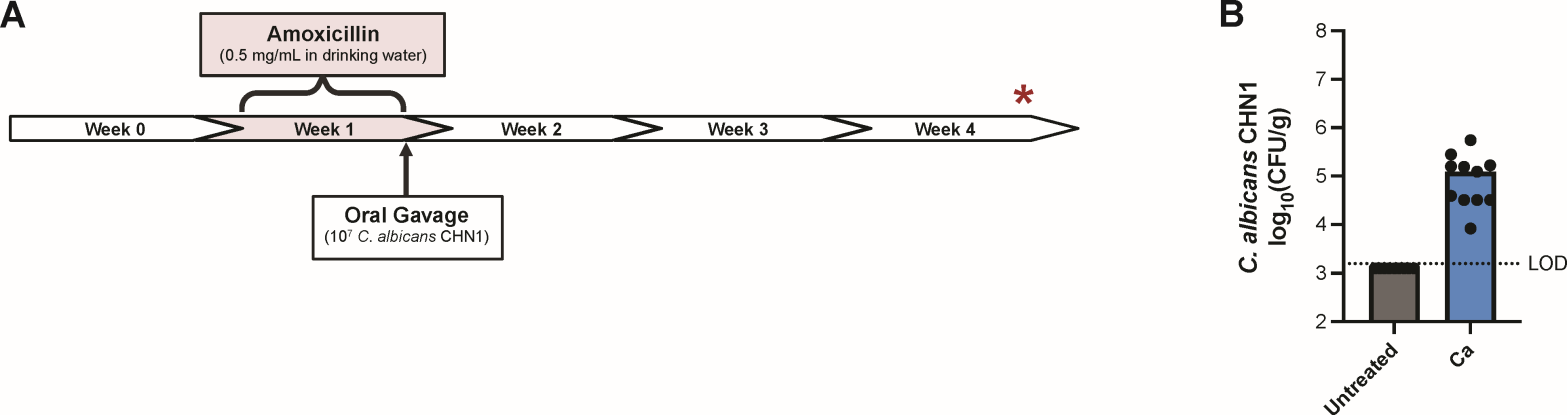
*Candida albicans* gastrointestinal colonization model. (**A**) Timeline of model used for *C. albicans* gastrointestinal colonization in mice. During the second week of the experiment (week 1), BALB/c mice were administered 0.5 mg/mL amoxicillin in their drinking water. At the end of the antibiotic regimen, mice were intragastrically inoculated with 10^7^ *C. albicans* CHN1 cells. Untreated mice did not receive any antibiotics. Study endpoint and sample collection are denoted by the red asterisk. Stomach tissue was collected from all mice for analysis at the end of week 4 (i.e., 3 weeks following the introduction of *C. albicans* CHN1) unless otherwise stated. (**B**) Gastric colonization levels of *C. albicans* CHN1 at the end of week 4, from two independent experiments (*N* = 20). Each circle represents the average of three plating replicates for a single mouse, and the bars correspond to median log CFU/g for each group. Ca, *C. albicans* CHN1-inoculated mice; LOD, Limit of detection.

**Fig 2:**
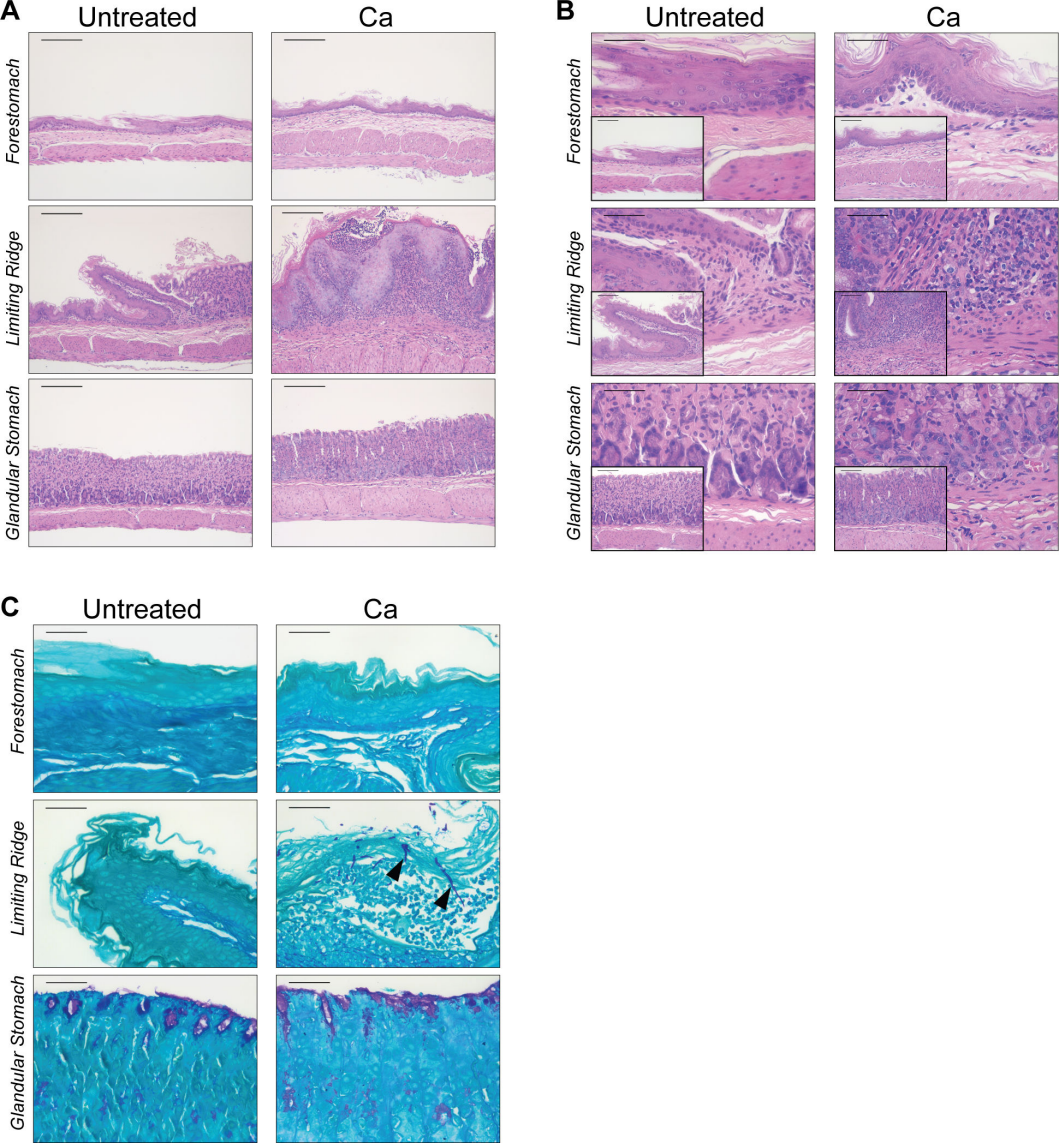
Stomach histology. (**A**) Hematoxylin and eosin (H&E)-stained stomach sections shown at low magnification (100×). Scale bars = 200 µm. Epithelial hyperplasia and submucosal edema were visible in the limiting ridge but were not evident in the forestomach or glandular stomach. (**B**) H&E-stained stomach sections shown at 400× (main panels) and 200× (insets). Scale bars = 100 µm (200× images) and 50 µm (400× images). Inflammatory infiltrates were observed in *C. albicans*-colonized stomachs but were concentrated at the limiting ridge. (**C**) Periodic acid–Schiff (PAS)-stained stomach sections shown at high magnification (400×). Scale bars = 50 µm. *C. albicans* cells (yeast or hyphae) were only visible in the limiting ridge region (examples of invasive hyphae are indicated by black arrowheads).

Our first objective was to determine whether *C. albicans* colonization of the murine stomach leads to an inflammatory response in the glandular region. *Candida albicans* strain CHN1 will stably colonize throughout the murine gastrointestinal tract (including the stomach) following a transient antibiotic-mediated dysbiosis (3, 30, 31). Thus, we administered the broad-spectrum antibiotic amoxicillin to mice in their drinking water for 1 week, followed by a single oral gavage with *C. albicans* CHN1 (Fig. 1A). We confirmed that *C. albicans* was colonizing the glandular stomach throughout the experiment by quantitative culture from homogenized stomach tissue on Sabouraud dextrose agar (SDA) (Fig. 1B), as well as PCR using *C. albicans* CHN1-specific primers on glandular stomach biopsies (data not shown). At 3 weeks post-inoculation (week 4), stomach sections were collected for histological analysis. Consistent with our previous observations (3), hyphal invasion and inflammatory cell infiltrates were evident in the limiting ridge of *C. albicans*-colonized mice (Fig. 2A and B). However, there was no histologically overt inflammation in the glandular stomach (Fig. 2B), and *C. albicans* cells (yeast or hyphal) were not visible at 400× (Fig. 2A) or 1,000× magnification (data not shown), indicating that invasion of the glandular stomach epithelium was minimal to non-existent. Collectively, these findings demonstrate that *C. albicans* CHN1 successfully colonizes the murine stomach and induces gastritis, but the inflammatory response is minimal in the glandular stomach mucosa.

### *C. albicans* colonization induces a minimal/low-level innate immune response in the glandular stomach

**Fig 3:**
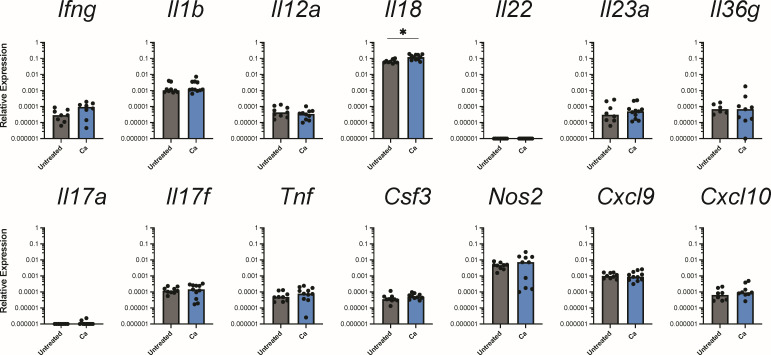
Expression of genes associated with type 1 and 3 immune responses. Relative expression of genes in the glandular stomach 3 weeks post-inoculation with *C. albicans* CHN1 (week 4) from two independent experiments (*N* = 20). Gene expression is shown relative to *Actb*. Each circle represents a single mouse with bars at the median relative expression for each group. ^*^P < 0.05.

Given the histological evidence of gastritis in the limiting ridge adjacent to the glandular stomach, our subsequent aim was to determine whether there was an immune response to *C. albicans* within the glandular stomach itself, even in the absence of overt inflammation. We carefully excised the glandular stomach between the limiting ridge and pyloric sphincter, making sure to exclude tissue from either of those two regions, and performed qPCR using a panel of 60 genes encompassing a wide range of mucosal immune responses in mice (Table S1). When we evaluated the expression of genes commonly associated with antifungal immunity, we saw significant upregulation of *Il18* but not *Ifng*, *Il1b*, nor *Il22* in the glandular stomach of *C. albicans*-colonized mice (Fig. 3). Additionally, only 2 out of the 11 *C*. *albicans*-colonized mice had a higher expression of *Il17a* (Fig. 3). When we evaluated gene expression at different timepoints in a single cohort time course experiment, we saw *Il17f* expression at week 3 in *C. albicans*-colonized mice, but *Il17a* expression was only moderately increased (3/11 mice at week 2 and 1/6 mice at week 4; Fig. S1). Thus, the host response to gastrointestinal colonization by *C. albicans* does not involve the induction of canonical type 3 antifungal immunity pathways in the glandular stomach.

**Fig 4:**
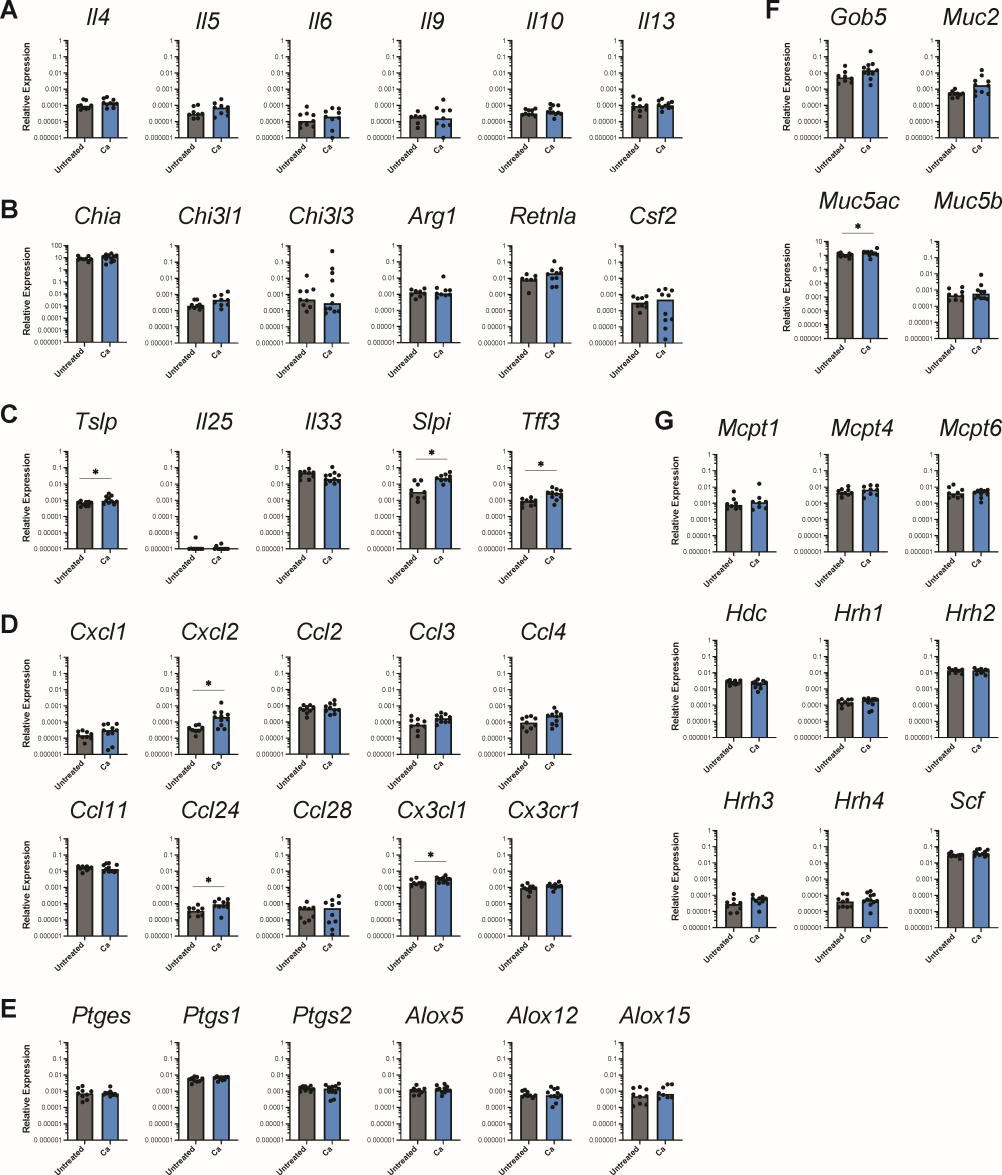
Expression of genes associated with type 2 immune responses and inflammation. Relative expression of genes in the glandular stomach 3 weeks post-inoculation with *C. albicans* CHN1 (week 4) from two independent experiments (*N* = 20). Gene expression is shown relative to *Actb*. Each circle represents a single mouse with bars at the median relative expression for each group. ^*^P < 0.05.

Next, we investigated whether *C. albicans* was inducing a type 2 immune response in the glandular stomach, since we had previously shown that gastrointestinal colonization with *C. albicans* CHN1 can promote the development of allergic diseases in the airways (21). Although the expression of type 2 cytokines *Il4*, *Il5*, and *Il13* appeared to be unaffected by *C. albicans* colonization (Fig. 4A), there was a significant upregulation of the cytokines *Tslp*, *Slpi*, and *Tff3* (Fig. 4C) and the major gastric mucin *Muc5ac* (Fig. 4F). We also saw a low-level expression of the chemokines *Cx3cl1*, *Cxcl2,* and *Ccl24* (involved in the recruitment of mononuclear leukocytes, neutrophils, and eosinophils, respectively) in *C. albicans*-colonized mice (Fig. 4D). We did not observe significant changes in the expression of type 2-inducible genes (Fig. 4B), eicosanoid biosynthetic pathways (Fig. 4E), or mast cell activation genes (Fig. 4G) in *C. albicans*-colonized mice. Overall, the pattern of gene expression observed in the glandular stomach is indicative of a minimal/low-level innate immune response to *C. albicans*.

### *C. albicans* colonization leads to changes in the expression of genes associated with epithelium integrity, signaling, and defense in the glandular stomach

**Fig 5:**
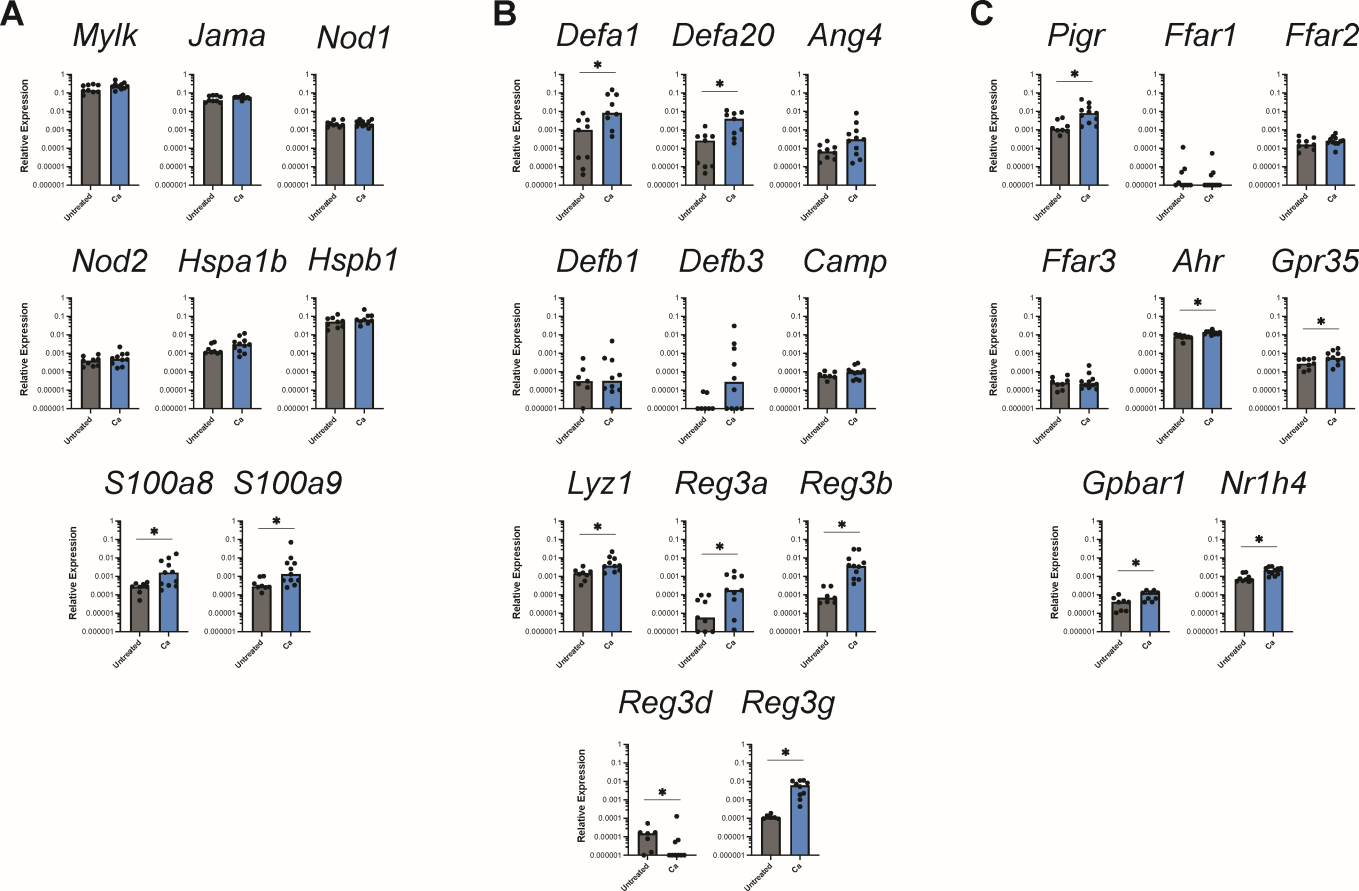
Expression of genes associated with epithelium responses. Relative expression of genes in the glandular stomach 3 weeks post-inoculation with *C. albicans* CHN1 (week 4) from two independent experiments (*N* = 20). Gene expression is shown relative to *Actb*. Each circle represents a single mouse with bars at the median relative expression for each group. ^*^P < 0.05.

In our histological analysis of the stomach, we noticed substantial submucosal edema and hyperplasia of the limiting ridge epithelium in *C. albicans*-colonized mice, which did not appear to extend into the glandular region (Fig. 2C). Given that we had seen upregulation of genes associated with innate immune responses despite a lack of overt inflammation, we investigated whether the glandular stomach epithelium was responding to *C. albicans* colonization even in the absence of any visible remodeling. We expanded our qPCR panel to include 28 additional genes with known roles in epithelium sensing and signaling, barrier integrity, and antimicrobial responses in the murine gastrointestinal tract (Table S2). The expression of *S100a8* and *S100a9* (encoding the protein complex calprotectin) was significantly higher in *C. albicans*-colonized mice compared to untreated mice (Fig. 5A), potentially reflecting an innate defense mechanism aimed at preventing tissue damage and fungal overgrowth. In line with the notion that *C. albicans* is triggering an antimicrobial response in the glandular stomach epithelium, we additionally saw upregulation of a number of antimicrobial peptides, including the alpha-defensins *Defa1* and *Defa20*, Reg3 proteins (*Reg3a*, *Reg3b*, and *Reg3g*), and lysozyme *Lyz1* (Fig. 5B). Notably, there was a significantly increased expression of *Ahr*, *Gpr35*, *Gpbar1*, and *Nr1h4* in the *C. albicans*-colonized mice (Fig. 5C), suggesting that the glandular stomach epithelium may be modulating the immune response to maintain tissue homeostasis. Consistent with the lack of glandular epithelium damage in the histology sections, there was no widespread upregulation of genes involved in barrier integrity or epithelial restructuring/repair (Fig. 5A). Taken together, our histological and gene expression analyses indicate that there is a low-level response by the epithelium and minimal inflammation in the glandular stomach, resulting in highly regionalized inflammation in the adjacent limiting ridge.

### *C. albicans* colonization of the stomach tract during food allergy

**Fig 6:**
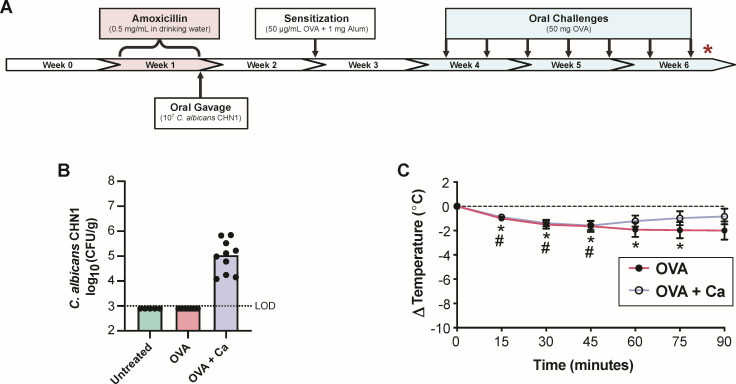
Food allergy induction and analysis of *C. albicans* gastrointestinal colonization. (**A**) Food allergy model timeline. Mice were intragastrically inoculated with *C. albicans* CHN1 according to the protocol detailed in Fig. 1A. At the end of week 2, mice were sensitized with a single intraperitoneal injection of 50 µg/mL OVA in 1 mg Alum. A total of seven OVA challenges were performed across weeks 4–6, wherein 50 mg OVA was given to the mice via oral gavage every 2–3 weeks. Stomach tissue was collected from all mice for analysis at the end of week 6 (after the final oral OVA challenge), as indicated by the red asterisk. (**B**) *C. albicans* CHN1 colonization levels in cecal tissue. Each circle represents the average of two plating replicates for a single mouse, and the lines correspond to median log CFU/g for each group. (**C**) Changes in body temperature following the final OVA challenge. Points correspond to mean temperature change ± SEM. Significant drops in temperature from baseline are denoted with asterisks for OVA mice (^*^P < 0.05) and hash marks for OVA + Ca mice (^#^*P* < 0.05).

**Fig 7:**
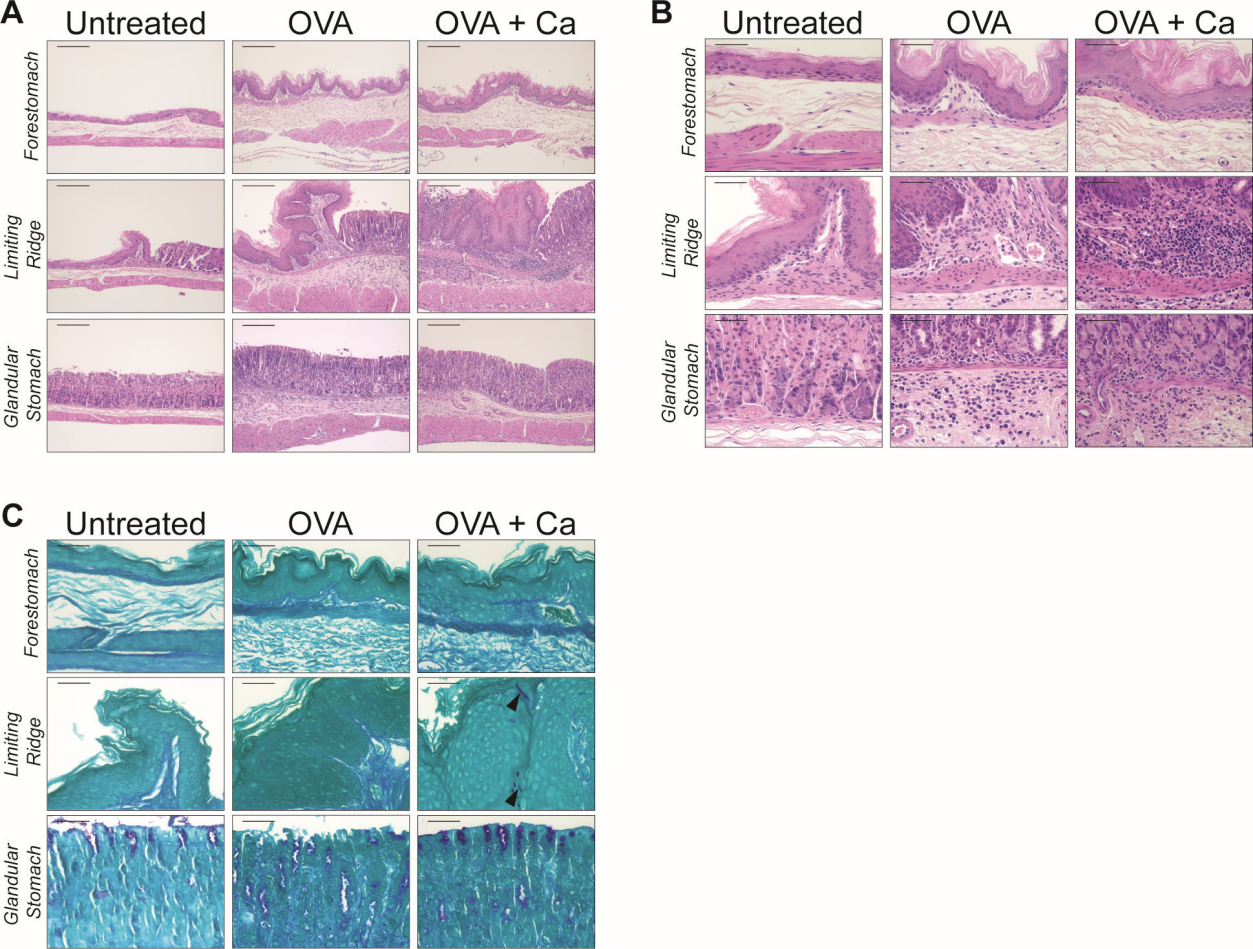
Stomach histology following OVA challenges. (**A**) H&E-stained stomach sections shown at low magnification (100×). Scale bars = 200 µm. Tissue remodeling was evident in OVA-challenged mice, which included submucosal edema and epithelial hyperplasia. (**B**) H&E-stained stomach sections shown at 400× (main panel) and 200× (insets). Scale bars = 100 µm (200× images) and 50 µm (400× images). OVA-challenged mice had a substantial influx of inflammatory cells in both the glandular stomach and limiting ridge, which were predominantly eosinophils, mononuclear cells, and neutrophils. (**C**) PAS-stained stomach sections shown at high magnification (400×). Scale bars = 50 µm. *C. albicans* cells (yeast or hyphae) were only visible in the limiting ridge region (examples of invasive hyphae are indicated by black arrowheads). Groups (from Fig. 6): OVA, sensitized and challenged with OVA; OVA + Ca, *C. albicans*-colonized, sensitized and challenged with OVA.

Pre-existing inflammation favors the growth of *C. albicans* in a host (22), and conversely, colonization by *C. albicans* has been shown to exacerbate inflammatory conditions in the gastrointestinal tract (6, 22, 32), including allergic diseases (33). Thus, our second objective was to determine (i) whether mucosal inflammation during a food allergic response occurs in the glandular stomach and (ii) if *C. albicans* colonization during food allergy induction promotes allergic inflammation in the glandular stomach mucosa or vice versa. We used the same protocol described earlier for gastrointestinal colonization by *C. albicans* CHN1, followed by the induction of experimental food allergy (34–37). BALB/c mice were systemically sensitized via intraperitoneal injection of OVA combined with the adjuvant aluminum hydroxide (Alum), then orally challenged with OVA every 2–3 days over the course of 3 weeks (Fig. 6A). After the seventh oral challenge, mice developed symptoms indicative of an anaphylactic response (Fig. 6C). We confirmed that *C. albicans* was colonizing the glandular stomach through the end of the experiment (week 6) via PCR, as well as the rest of the gastrointestinal tract by quantitative culture of homogenized cecal tissue (Fig. 6B). Once again, despite being detectable throughout the gastrointestinal tract after the OVA challenges, *C. albicans* hyphal invasion was observed only in the limiting ridge and not glandular stomach (Fig. 7A).

### Inflammatory cell recruitment and tissue remodeling occur in the glandular stomach following oral OVA challenges but are not further augmented by *C. albicans* colonization

Upon closer examination of the glandular stomach in histological sections from the OVA-challenged mice, we observed a substantial influx of inflammatory cells (neutrophils, eosinophils, and mononuclear cells) in the submucosa and lamina propria, which were notably absent in the untreated mice (Fig. 7B). There were also pronounced changes in the overall architecture of the glandular stomach epithelium, including submucosal edema, remodeling of the underlying muscularis, and epithelial cell hyperplasia (Fig. 7C). Overall, these data indicate that oral OVA challenges in sensitized mice cause robust inflammation that is accompanied by remodeling of the gastric epithelium.

When we examined the glandular stomach histology from OVA-challenged mice that were colonized with *C. albicans*, we did not see any appreciable differences in the magnitude of the inflammatory response or the types of inflammatory infiltrates compared to the uncolonized OVA-challenged group (Fig. 7B). Colonization by *C. albicans* did not lead to additional changes in the structure of the glandular stomach epithelium (Fig. 7C). Altogether, there is histologically evident inflammatory cell recruitment and epithelium remodeling in response to food allergy induction in the glandular stomach.

### Oral OVA challenges trigger a type 2 inflammatory response in the glandular stomach of food allergic mice

**Fig 8:**
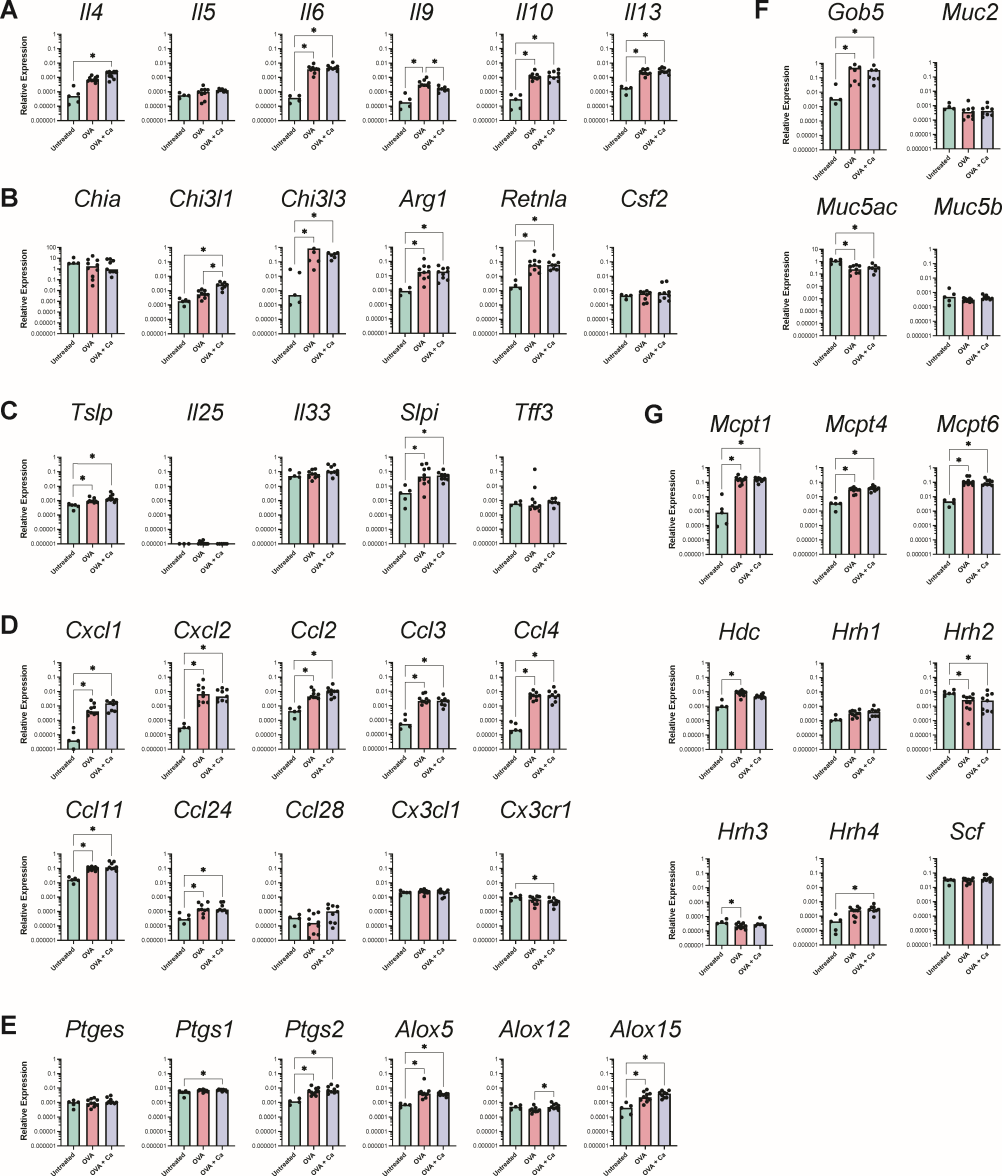
Expression of genes associated with type 2 immunity and inflammation in the glandular stomach after the final oral OVA challenge. Relative expression of genes in the glandular stomach at the end of week 6 from two independent experiments (*N* = 25). Groups (from Fig. 6): OVA, sensitized and challenged with OVA; OVA + Ca, *C. albicans*-colonized, sensitized and challenged with OVA. Gene expression is shown relative to *Actb*. Each circle represents a single mouse with bars at the median relative expression for each group. ^*^P < 0.05.

**Fig 9:**
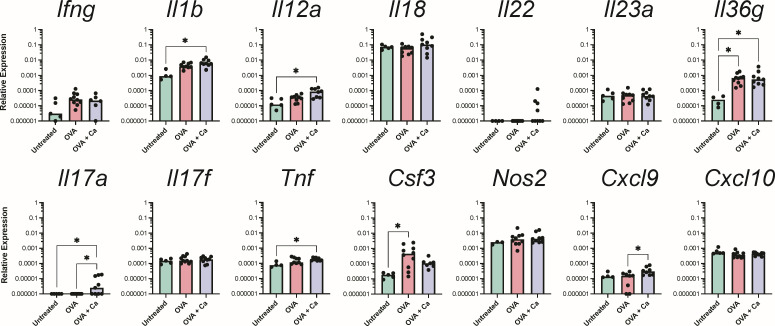
Expression of genes associated with type 1 and 3 immunity in the glandular stomach after the final oral OVA challenge. Relative expression of genes in the glandular stomach at the end of week 6 from two independent experiments (*N* = 25). Groups (from Fig. 6): OVA, sensitized and challenged with OVA; OVA + Ca, *C. albicans*-colonized, sensitized and challenged with OVA. Gene expression is shown relative to *Actb*. Each circle represents a single mouse with bars at the median relative expression for each group. ^*^P < 0.05.

Next, we wondered if the type 2-polarized immune response characteristic of food allergy and anaphylaxis in the intestinal tract was also occurring in the glandular stomach, as our histological analysis seemed to suggest. There was a significant upregulation of most of the genes encoding type 2 cytokines (Fig. 8A), type 2-inducible molecules (Fig. 8B), and *Tslp* and *Slpi* (Fig. 8C) in the glandular stomach of OVA-challenged mice relative to untreated mice. There was no significant induction of genes in the type 1 or type 3 pathways in OVA-challenged food allergic mice (Fig. 9).

We next examined the expression of genes associated with mast cell responses and mucus production. All three major murine mast cell proteases (*Mcpt1*, *-4*, and *-6*) showed a significant increase in expression in all the OVA-challenged mice compared to untreated mice, as well as histidine decarboxylase (*Hdc*) (Fig. 8G). Additionally, the expression of the goblet cell regulator *Gob5* was higher in OVA-challenged mice, while *Muc5ac* (which encodes mucin that coats the gastric epithelium) was decreased in OVA-challenged mice (Fig. 8F). In our analysis of genes involved in the biosynthesis of eicosanoids, the cyclooxygenase *Ptgs2* and the lipoxygenases *Alox5* and *Alox15* also demonstrated a significant increase in expression in response to the OVA challenges in food allergic mice (Fig. 8E). Altogether, these results indicate that there is a type 2-polarized immune response to OVA challenges in the glandular stomach, including activation of mast cells and goblet cells.

### *C. albicans* colonization leads to upregulation of innate and type 3, but not type 2, inflammation in the glandular stomach

To determine whether colonization with *C. albicans* CHN1 augments type 2 immune response to oral OVA challenges in the glandular stomach, we assessed gene expression in *C. albicans*-colonized mice that had received the oral OVA challenges. The *C. albicans*-colonized mice had similar expression levels to the uncolonized OVA mice for each gene associated with type 2 immunity (Fig. 8A through C), and we did not see any additional upregulation of genes associated with mast cell responses (Fig. 8G), mucus production (Fig. 8F), or eicosanoid biosynthesis (Fig. 8E). However, *C. albicans* colonization augmented innate inflammatory responses and induced the expression of IL-17 in the glandular stomach during allergic inflammation. There was a significantly higher expression of the innate inflammatory cytokines *Il1b*, *Il12a*, and *Tnf* (Fig. 9) and significant upregulation of *Il17a* in the *C. albicans*-colonized food allergic mice compared to both the untreated and uncolonized OVA-challenged mice (Fig. 9).

### Oral OVA challenges lead to an upregulation of pro-inflammatory chemokines in the glandular stomach that is not affected by *C. albicans* colonization

We next analyzed chemokine expression in the glandular stomach. Consistent with the observed recruitment of neutrophils, eosinophils, and mononuclear cells in the glandular stomach histology sections (Fig. 7B), there was increased expression of the eosinophil chemoattractants *Ccl11* and *Ccl24*, as well as increased expression of the mononuclear leukocyte chemokines *Ccl2*, *-3*, and *-4* following the OVA challenges (Fig. 8D). There was also a significant induction of genes associated with neutrophil activation such as *Ccxl1*, *Cxcl2*, *Csf3*, and *Il36g* in response to the OVA challenges in food allergic mice (Fig. 8D and 9). *C. albicans* colonization did not further augment chemokine expression in the glandular stomach during a food allergy response (Fig. 8D).

### Oral OVA challenges induce changes in the expression of genes involved in barrier integrity and signaling in the glandular stomach, which are not affected by *C. albicans* colonization

**Fig 10:**
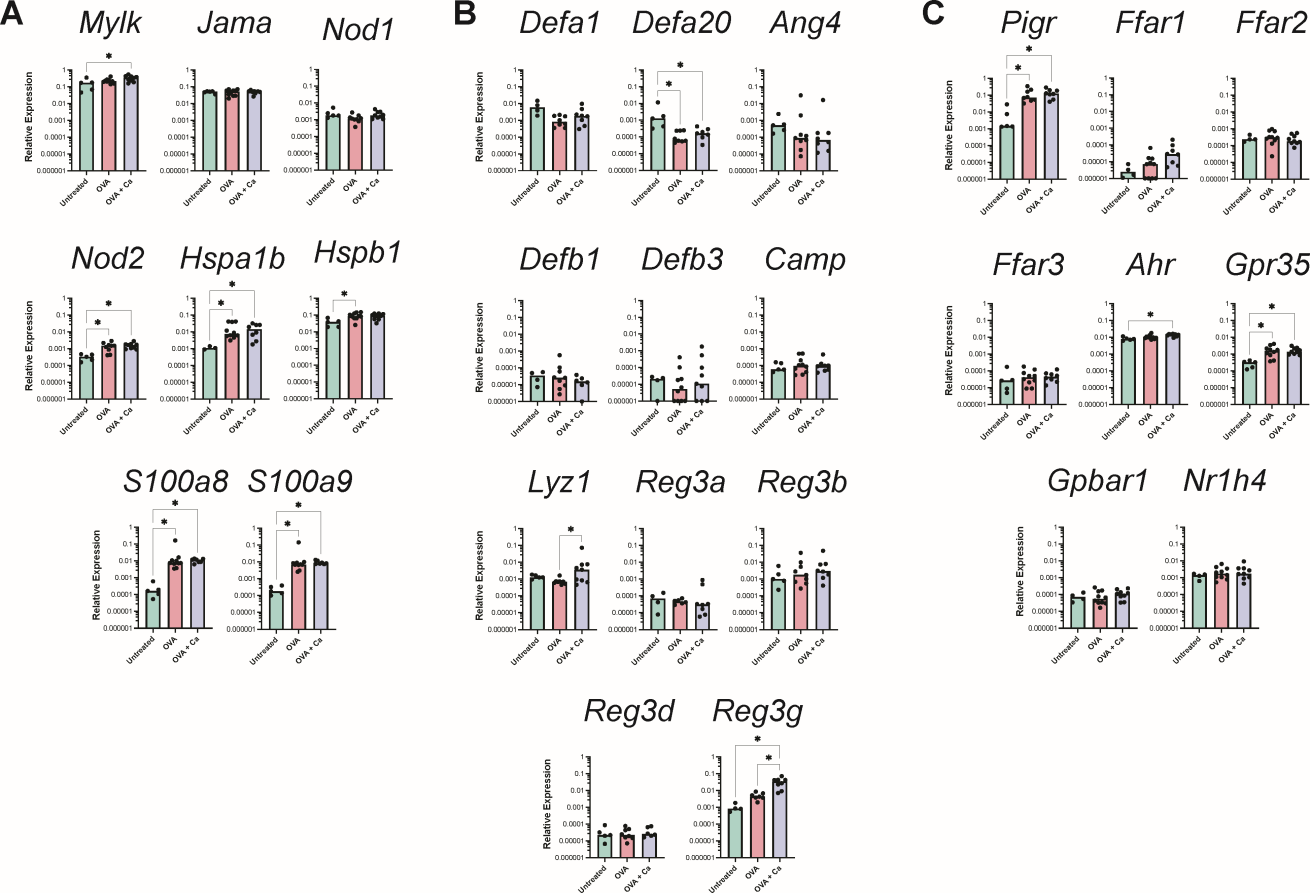
Expression of genes associated with epithelium responses in the glandular stomach after the final oral OVA challenge. Relative expression of genes in the glandular stomach at the end of week 6 from two independent experiments (*N* = 25). Groups (from Fig. 6): OVA, sensitized and challenged with OVA; OVA + Ca, *C. albicans*-colonized, sensitized and challenged with OVA. Gene expression is shown relative to *Actb*. Each circle represents a single mouse with bars at the median relative expression for each group. ^*^P < 0.05.

Our histology data showing glandular stomach epithelium remodeling also prompted us to investigate the expression of genes associated with epithelium integrity, sensing, and signaling. We first evaluated the expression of a set of genes involved in maintaining the epithelial barrier and sensing cellular damage. Consistent with the histological changes, a number of these genes were significantly upregulated in OVA-challenged mice relative to untreated mice (Fig. 10A through C). In particular, *Gpr35* (which is involved in the protection of the epithelium during inflammation) and the heat shock proteins *Hspa1b* and *Hspb1* (which promote gastric mucosal protection during stress) were all highly expressed in the glandular stomach of OVA-challenged mice (Fig. 10A and C). Finally, we observed that the expression of many of the genes encoding antimicrobial peptides was not affected by the OVA challenges in food allergic mice (Fig. 10B). The expression of *Reg3g*, *S100a8*, and *S100a9* was significantly elevated in OVA-challenged mice, while that of *Defa20* was significantly decreased. The polymeric Ig receptor (*Pigr*) was also more highly expressed in the glandular stomach of food allergic mice (Fig. 10C). To our surprise, *C. albicans* colonization did not significantly affect the expression of these genes in OVA-challenged food allergic mice (Fig. 10A through C). Altogether, these results indicate that the oral OVA challenges elicit an inflammatory response in the glandular stomach mucosa that drives changes in the expression of genes associated with epithelium integrity, sensing, and signaling that is independent of the presence or absence of *C. albicans*.

## DISCUSSION

Our study aimed to evaluate the localized host reaction in the glandular stomach when colonized by *C. albicans*. We also sought to determine whether a food allergy in mice could augment *C. albicans* pathogenesis and inflammation of the stomach or, conversely, if *C. albicans* colonization could intensify the allergic response. In mice, *C. albicans*-induced gastritis is almost exclusively concentrated at the limiting ridge—the demarcation line segregating the forestomach from the hindstomach (glandular stomach). An intriguing observation reported by many laboratories, and replicated in our current study, is that during gastric infection, the glandular stomach mucosa remains visually unmarked by inflammation. This raises the possibility that gastric inflammation due to *C. albicans* infection may be focused at the limiting ridge, suggesting a localized host response. We observed an increased expression of *Il18* in the glandular stomach post-colonization by *C. albicans*, yet no significant changes were noted for other cytokines related to type 1, 2, or 3 immune responses. We observed an increase in the expression of antimicrobial peptides, such as the alpha-defensins *Defa1* and *Defa20*, Reg3 proteins, lysozyme *Lyz1*, and calprotectin (*S100a8* and *S100a9*). In mice with an induced food allergy to OVA, significant type 2 immune responses, inflammatory cell recruitment, epithelium signaling, and tissue restructuring were observed following oral challenge with OVA. Colonization by *C. albicans* did not further augment the robust type 2 inflammatory responses in the glandular stomach of food allergic mice, but it did result in the upregulation of *Il1b, Il12a, Tnfa,* and *Il17a* in this region of the stomach. Thus, while *C. albicans* colonization of the glandular stomach induces minimal inflammation, it does stimulate increased antimicrobial peptide expression, and food allergy induction elicits a strong type 2 inflammatory response that extends into the glandular stomach and promotes innate and type 3 inflammation in response to *C. albicans*.

In agreement with our previous work (3), we demonstrated that *C. albicans* causes gastritis and assumes an invasive hyphal form at the limiting ridge of the murine stomach. This is consistent with decades of work with numerous *C. albicans* strains in multiple mouse strains demonstrating that gastritis is almost exclusively localized to the limiting ridge (3, 14–19). Many groups have also noted that the immune response to *C. albicans* is highly context-dependent and differs dramatically between tissue sites (38–42). Romani and colleagues have published a number of studies analyzing the murine host response to gastric colonization by *C. albicans*, resulting in a model that describes the host response to *C. albicans* colonization in the stomach. Their data support a paradigm in which type 1 and regulatory responses to *C. albicans* colonization are protective, type 2 responses do not play a role, and type 3 responses promote gastric inflammation and a phenotypic switch from commensal to pathogenic (15, 24, 25, 43–48). This latter process involves cytokine induction (IL-23, IL-6, TGF-β, and IL-17), IL-1R1 signaling, NLRP3 inflammasome activation, augmentation of neutrophil-mediated pathology, and downregulation of IL-22. The Dectin-1 receptor is required to control gastritis and colonization in C57BL/6 but not BALB/c mice (49). Both mouse strains have higher levels of TNFα, IL-6, IL-17A, and IL-17F in whole stomach homogenates after infection, illustrating that the type 3 model of gastric mucosal inflammation and *C. albicans* pathogenesis is applicable in both C57BL/6 and BALB/c mice.

In this and previous work, we have demonstrated that *C. albicans* CHN1 colonization of the murine stomach results in highly regionalized gastritis of the limiting ridge. Numerous *C. albicans* strains are capable of colonizing the murine stomach and causing gastritis (14–19, 27). This may be a unifying feature of *C. albicans* biology across genotypic variants, similar to the capacity of all strains to cause disease in a *Galleria mellonella* infection model, despite strain divergence in the genome or murine intestinal colonization phenotype (31, 50–52). In a side-by-side study, we have previously demonstrated that *C. albicans* strains CHN1 and SC5314 are both able to induce gastric inflammation that is exclusively localized to the limiting ridge and accompanied by hyphal transformation and an influx of neutrophils and mononuclear cells (3), consistent with the decades of study of other *C. albicans* strains in mice.

The intestinal bacterial microbiota has been implicated in the pathophysiology of food allergy and other allergic diseases (27, 37, 53–60). However, studies on the mechanisms and pathophysiology of food allergy development have largely excluded the gastric mucosa from investigation, yet the dietary antigen structure (e.g., size, solubility, and stability to digestive processes during transit through the gastrointestinal tract) is known to greatly contribute to the allergenic potential of foods (61). For example, gastric acid suppression was found to promote allergic disease in both animal models and humans (62, 63), underscoring the importance of the stomach and its highly acidic environment in promoting oral tolerance through food antigen processing. Oliveira and colleagues demonstrated that palmitoyl residues coupled to OVA prevent oral tolerance to OVA (64, 65) and promote OVA retention within the stomach (66), suggesting that the pattern of antigen proteolysis by gastric enzymes is central to the generation of epitopes with either immunogenic or immunosuppressive properties. An earlier study in rats reported that an IgE-mediated food allergic response to OVA can occur in the stomach, with elevated levels of intraluminal histamine, mast cell degranulation, and rat mast cell protease II, as well as a significant increase in gastric acid secretion and disrupted stomach motility (67). Our current study begins to address this major gap in knowledge surrounding mucosal immune responses in the stomach during food allergy. We have demonstrated here that a potent food allergic response occurs in the glandular stomach following oral challenges in sensitized mice, with induction of a polarized type 2 immune response, including significant increases in the expression of type 2 cytokines, chemokines, and mast cell proteases, as well as alterations in the epithelium.

We did not see an induction of a type 2 immune response due to *C. albicans* colonization alone, nor increased expression of the mammalian chitinase *Chia*. A recent report showing that high levels of dietary chitin can cause gastric distension, trigger a type 2 immune response in mice, and lead to increased endogenous chitinase activity (68) raised the possibility that *C. albicans* cell wall chitin (or indeed dietary chitin derived from chow) could be driving a type 2 response in our mouse model of food allergy. Thus, our findings were unexpected. It has also been reported that gastrointestinal colonization with *C. albicans* inhibits the induction of humoral immune tolerance and leads to mast cell degranulation in the stomach of OVA-challenged mice, as well as increased gut permeability (15, 26, 27, 49, 69). We observed only minimal induction of *Il17a* and *Il17f* in the glandular stomach during *C. albicans* colonization in the absence of an underlying inflammatory response.

However, induction of a food allergy to OVA led to significant upregulation of *Il17a* along with elevated expression of *Il1b* and *Il12a*, indicating that allergic inflammation augments the inflammatory response to *C. albicans* in the glandular stomach. We have recently completed a series of studies of the inflammatory response profile in the limiting ridge and have seen robust induction of *Il17a* and *Il17f* (Zeise et al., data not shown), which is similar to previous reports of type 3 response induction in whole stomach tissue (15, 24, 25, 43–48). *C. albicans* colonization of other mucosal sites can lead to higher levels of protective IL-17A in the colon (70) and oral cavity (71). Elevated IL-17 and IL-9/mast cell signaling have been associated with *C. albicans* pathogenicity during inflammatory bowel disease and celiac disease, respectively (38, 72). One intriguing finding from our studies is that the induction of a food allergic response resulted in the reversal of the *C. albicans*-induced antimicrobial peptide expression. We hypothesize that this could be the result of (i) alterations in the epithelium as a result of the robust type 2 inflammatory response, (ii) induction of innate immunity and type 3 immunity that is a sufficient antimicrobial response to limit microbial invasion, or (iii) additional alterations in the bacterial microbiota of the stomach during the allergic response.

Given that underlying type 2 inflammation has been linked to increased fungal growth and dissemination (73), it is intriguing that food allergy induction did not trigger the spread of *C. albicans* hyphal invasion to the glandular stomach or amplify the type 2 immune response. Since commensal colonization is a prerequisite for opportunistic infection, there have been a number of studies investigating the genetic circuitry that enables *C. albicans* to adapt to a host environment and subsequently cause infection (74–77). Notably, the transcriptional networks regulating the commensal and pathogenic lifestyle of *C. albicans* are now known to be tightly intertwined, with many genes involved in hyphal morphogenesis and virulence also being important for colonization and stress response (74, 75). Whether or not *C. albicans* becomes virulent is dependent on a number of environmental factors such as the bacterial microbiota, host diet, and immune milieu (2). The “damage response framework” has been used to describe *C. albicans* pathogenesis and its distinct interactions across different host environments, highlighting the host itself as an equally important factor in the balance between commensalism and pathogenicity (40, 78, 79). In our current study, we present evidence that the host response to *C. albicans* within the murine stomach is compartmentalized, suggesting that in the absence of other underlying gastric inflammation, the limiting ridge is a site of mucosal infection and the glandular stomach is a site of commensal colonization by *C. albicans*.

## MATERIALS AND METHODS

### Animals and housing

Eight- to twelve-week-old BALB/c mice were housed under specific-pathogen-free conditions in enclosed filter-top cages at the University of Michigan (Ann Arbor, MI), with food and sterile water provided *ad libitum*. Mice in a given experiment were all housed in the same room in our animal facility.

### Antibiotic treatment and *C. albicans* gastric inoculation

Amoxicillin (0.5 mg/mL; Sigma-Aldrich, St. Louis, MO) was administered orally to mice in their drinking water for 5 days prior to *Candida albicans* CHN1 colonization. Antibiotic-containing drinking water was replaced with sterile water after 7 days. Prior to gavage, *C. albicans* strain CHN1 was grown in Sabouraud dextrose broth (Difco, Detroit, MI) to the stationary phase in a shaking flask at 37°C. Cultures were washed in sterile non-pyrogenic saline, counted using a hemocytometer, and diluted to 2 × 10^8^ cells/mL. Mice were intragastrically inoculated with *C. albicans* CHN1 (10^7^ cells in 50 µL) using a 24-gauge feeding needle attached to a 1-mL syringe. The syringe containing *C. albicans* CHN1 was mounted on a Stepper repetitive pipette (Tridak, Brookfield, CT) to deliver a consistent amount of inoculum to each mouse. To verify the CFU delivered to the mice, the inoculum was serially diluted and plated on SDA (Difco).

### Murine model of food allergy

Mice were sensitized with 50 µg endotoxin-free OVA mixed with 1 mg Alum adjuvant (InvivoGen, San Diego, CA) via intraperitoneal injection (Fig. 6A). Two weeks later, mice were intragastrically challenged with 50 mg OVA (Sigma-Aldrich, Darmstadt, Germany) dissolved in sterile saline. A total of seven oral challenges were given every 2–3 days, and mice were fasted for 3–4 h prior to each challenge. Baseline rectal temperatures were taken prior to the seventh OVA challenge, and body temperatures were recorded every 15 min with a rectal probe (Physitemp Instruments, Clifton, NJ) for 90 min post-challenge, and anaphylaxis was defined as a temperature drop >0.5°C.

### Sample collection

Mice were euthanized by CO_2_ asphyxiation. Blood was collected in tubes containing SST clotting gel (BD Biosciences, Franklin Lakes, NJ) and centrifuged to isolate serum. Stomachs were removed and opened along the greater curvature, then rinsed in phosphate-buffered saline to remove the contents. Glandular stomach sections were collected for RNA and DNA. The RNA sections were submerged in RNAlater (ThermoFisher, Waltham, MA) and stored at −80°C, while DNA sections were snap-frozen in liquid nitrogen before being stored at −80°C. Histological sections were taken from the outer curvature of the stomach to encompass the forestomach, limiting ridge, and glandular stomach and were fixed with 4% buffered formalin and embedded in paraffin. These samples were then stained with hematoxylin and eosin for the detection of inflammatory infiltrates and Periodic acid–Schiff for the detection of fungal cells. The remaining stomach tissue was homogenized in sterile water, serially diluted, and cultured on SDA supplemented with 0.1 mg/mL cefoperazone (for enumeration of *C. albicans* CFUs).

### RNA isolation and gene expression analysis

Stomach sections were removed from RNAlater storage solution then homogenized and processed in a TRIzol reagent (Life Technologies, Carlsbad, CA) according to the manufacturer’s instructions. Isolated RNA was then purified using the RNeasy Mini Kit (Qiagen, Hilden, Germany) according to the manufacturer’s instructions. The concentration and purity of the RNA were evaluated using a nanodrop instrument (ThermoFisher, Waltham, MA) and Agilent Bioanalyzer (Agilent, Santa Clara, CA), respectively. cDNA was synthesized from the purified RNA using the RT^2^ first strand kit (Qiagen). Gene expression levels were measured using a custom RT^2^ Profiler PCR Assay (Qiagen), which includes genes associated with various aspects of the innate and adaptive immune system and three housekeeping genes (*Actb*, *Hprt*, and *Tbp*) as described previously (37). We have included tables listing the genes in our custom-designed RT^2^ Profiler qPCR panels (Tables S1 and S2). qPCR was run using Roche LightCycler 480 (Roche, Basel, Switzerland), and relative expression to *Actb* (which gave the most reproducible results and was therefore used for normalization) was calculated as 2^−ΔCt^. The *Actb* Ct values and gene ΔCt values for all our samples (following cross-plate normalization) that were used to assemble our figures and perform statistical analyses are included as a spreadsheet in the supplemental material for this paper.

For data reproducibility, the custom-designed RT^2^ Profiler qPCR panels are readily commercially available, and the qPCR panel quality control and design information is provided by the vendor (https://www.qiagen.com): “The RT² qPCR Primer Assays use SYBR Green-based quantitative real-time PCR technology, which provides a sensitive and reliable tool for gene expression analysis. Each assay utilizes a proprietary and experimentally verified algorithm for the design of gene-specific qPCR primers with uniform PCR efficiency and amplification conditions. Each lot of assay is further wet-bench-tested for real-time PCR performance for specificity and amplification efficiency. Amplification of a single product of the correct size with high PCR efficiency (>90%) is guaranteed when the assays are used with RT² SYBR Green qPCR Mastermixes.”

### Statistical analysis

Statistical significance testing was done using GraphPad Prism 10 software or R. For all statistical analyses, significance was set at *P* < 0.05. Student’s *t*-test with a Bonferroni correction for multiple comparisons was used to identify significant differences in body temperature from baseline for OVA and OVA + Ca mice. For gene expression data, outlier data points were identified and removed from the data set using the median absolute difference method (80) with some modifications. A Mann–Whitney *U*-test was used to determine statistical significance for pairwise comparisons between *C. albicans*-colonized mice and untreated mice, and a Kruskal–Wallis non-parametric analysis of variance was used for gene expression data from the food allergy model.

## Data Availability

Sequences used in 16S rRNA analyses are available on the NCBI Sequence Read Archive. The data repository can be found at https://www.ncbi.nlm.nih.gov/ under the accession number PRJNA1089162.

## SUPPLEMENTAL MATERIAL

Fig. S1 mbio.02113-24-s0001.tif Time course analysis of genes associated with type 1 and 3 immunity in the glandular stomach.

Fig. S2 mbio.02113-24-s0002.tif Time course analysis of genes associated with type 2 immunity and inflammation in the glandular stomach.

Fig. S3 mbio.02113-24-s0003.tif Time course analysis of genes associated with epithelium responses in the glandular stomach.

Tables S1 and S2 mbio.02113-24-s0004.xlsx Ct values.

ASM does not own the copyrights to Supplemental Material that may be linked to, or accessed through, an article. The authors have granted ASM a non-exclusive, world-wide license to publish the Supplemental Material files. Please contact the corresponding author directly for reuse.
