# Supplementary figures and images for: Profiling inflammatory outcomes of Candida albicans colonization and food allergy induction in the murine glandular stomach
Source: mBio. 2024 Sep 30;15(11):e02113-24. doi: 10.1128/mbio.02113-24 (PMC11559088; doi:10.1128/mbio.02113-24)

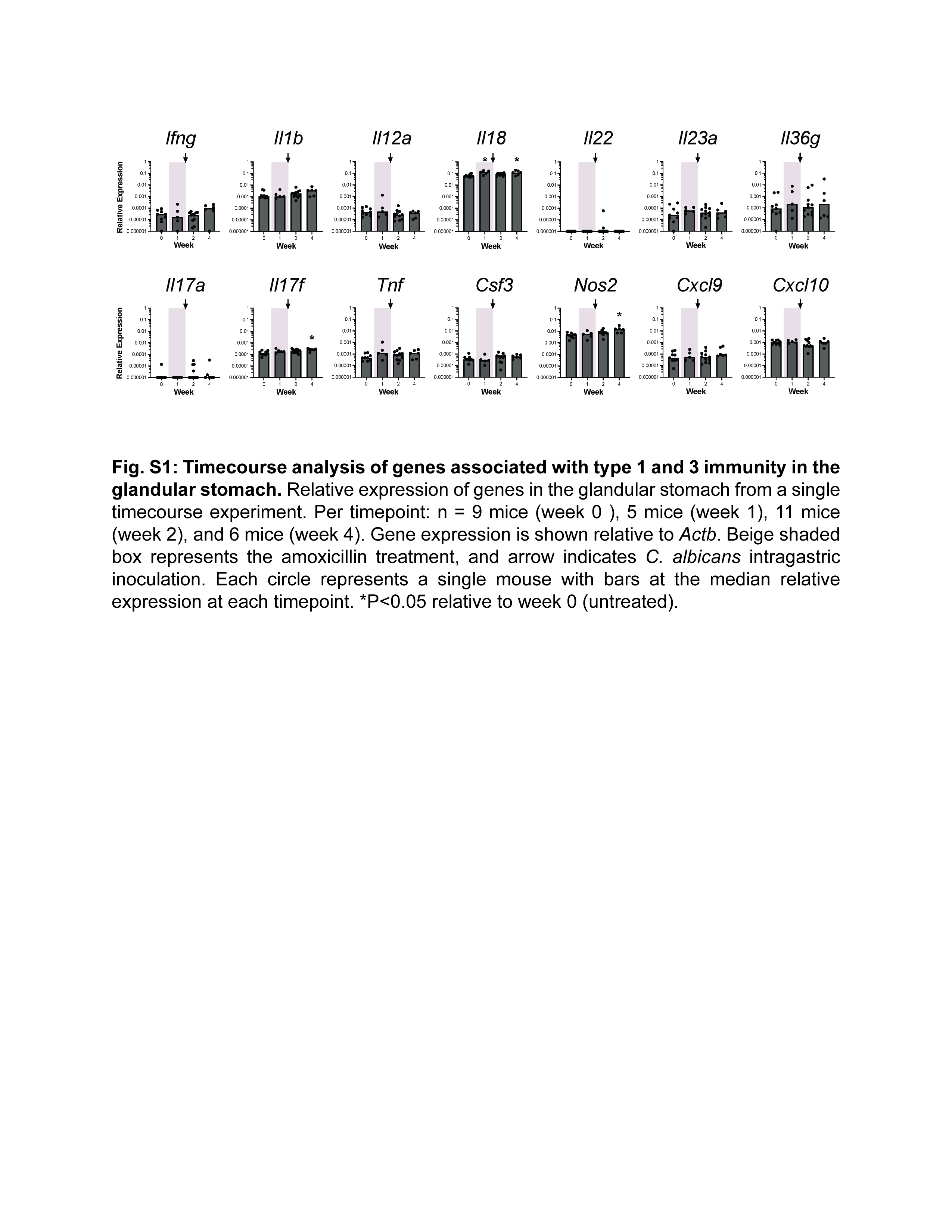

Supplement: Fig. S1 — Time course analysis of genes associated with type 1 and 3 immunity in the glandular stomach. [file mbio.02113-24-s0001.tif]

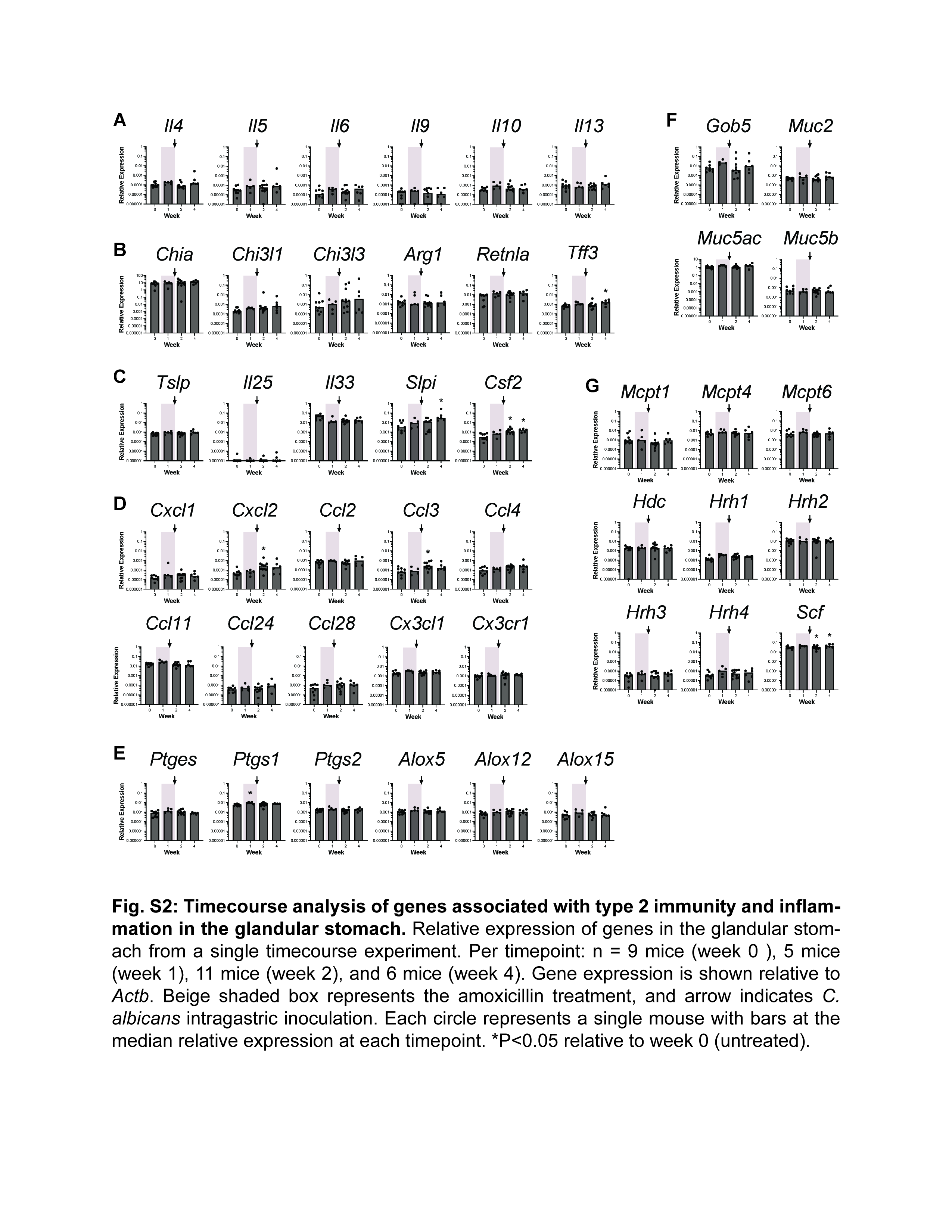

Supplement: Fig. S2 — Time course analysis of genes associated with type 2 immunity and inflammation in the glandular stomach. [file mbio.02113-24-s0002.tif]

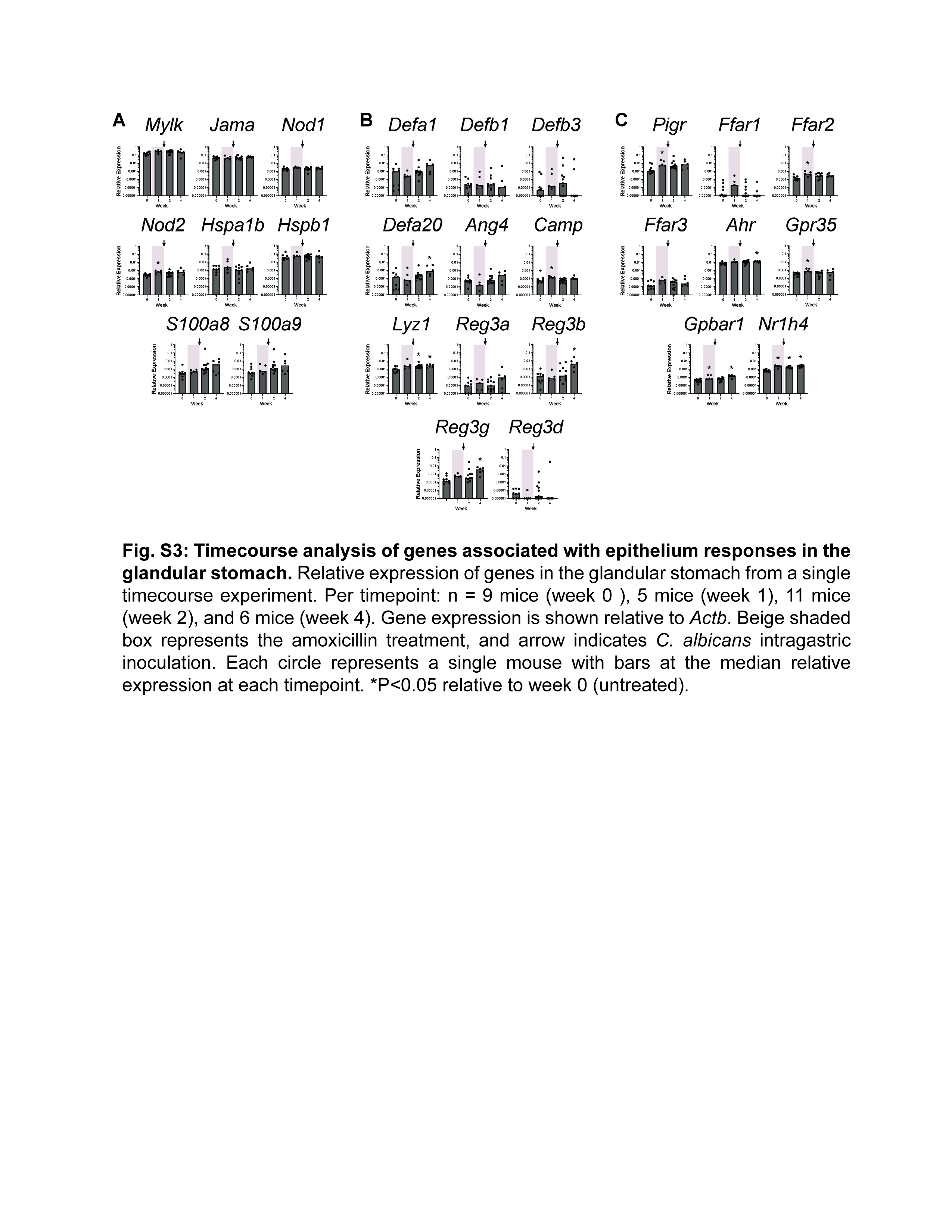

Supplement: Fig. S3 — Time course analysis of genes associated with epithelium responses in the glandular stomach. [file mbio.02113-24-s0003.tif]
